# Discovery and Characterization of a Novel Cyclic Peptide That Effectively Inhibits Ephrin Binding to the EphA4 Receptor and Displays Anti-Angiogenesis Activity

**DOI:** 10.1371/journal.pone.0080183

**Published:** 2013-11-12

**Authors:** Xiaofeng Han, Yan Xu, Yilei Yang, Jingle Xi, Wang Tian, Srinivas Duggineni, Ziwei Huang, Jing An

**Affiliations:** 1 Department of Pharmacology, SUNY Upstate Medical University, Syracuse, New York, United States of America; 2 SUNY Upstate Cancer Research Institute, Department of Pharmacology, State University of New York, Syracuse, New York, United States of America; 3 Department of Oncology, Nanfang Hospital, Southern Medical University, Guangzhou, China; University of Akron, United States of America

## Abstract

The EphA4 receptor tyrosine kinase regulates a variety of physiological and pathological processes during neural development and the formation of tumor blood vessels; thus, it represents a new and promising therapeutic target. We used a combination of phage peptide display and computer modeling/docking approaches and discovered a novel cyclic nonapeptide, now designated TYY. This peptide selectively inhibits the binding of the ephrinA5 ligand with EphA4 and significantly blocks angiogenesis in a 3D matrigel culture system. Molecular docking reveals that TYY recognizes the same binding pocket on EphA4 that the natural ephrin ligand binds to and that the Tyr3 and Tyr4 side chains of TYY are both critical for the TYY/EphA4 interaction. The discovery of TYY introduces a valuable probe of EphA4 function and a new lead for EphA4-targeted therapeutic development.

## Introduction

 The Eph receptors belong to the subfamily of receptor tyrosine kinases (RTKs) [1-4]. To date, 16 Eph receptors have been identified; these fall into two subclasses that show similar overall structure but have different binding affinities for the ephrin ligands [2,3]. The EphA4 receptor has a number of important functions in the vascular and neural development occurring during embryogenesis, postnatal tissue repair, and tumorigenesis [3,5-7]. It is expressed in neurons, where it promotes the repulsive guidance of developing axons [8,9]. Mice lacking the EphA4 exhibit pronounced axonal regeneration and functional recovery following spinal cord injury [9,10]. Inhibition of EphA4 by soluble protein inhibitors such as ephrin-A5-Fc, EphA4-Fc, or peptides induces axonal regeneration following spinal cord injury and reduces ischemia-induced apoptotic neuronal cell death [11,12]. In humans with amyotrophic lateral sclerosis (ALS), EphA4 expression inversely correlates with disease onset and survival. Knockdown of EphA4 is able to rescue the axonopathy induced by proteins that cause ALS [13]. Therefore, inhibition of the EphA4-ephrin interaction can be a useful strategy for promoting axon regeneration in the nervous system [14,15].

 A potential role for EphA4 in human cancer is also receiving increasing attention. Altered expression patterns of EphA4/ephrins are correlated with tumor behaviors, such as invasiveness, vascularization, and metastatic potential. Overexpression of EphA4 is observed in many human cancers, including prostate, breast, gastric, and pancreatic tumors [16-20]. High levels of EphA4 RNA expression are correlated significantly with reduced overall survival of cancer patients [21].

 EphA4 binds to both type A ephrins and most type B ephrins, but it also forms a hetero receptor complex with fibroblast growth factor receptor (FGFR). Signaling pathways mediated by EphA4 and FGFR play critical roles in promoting the proliferation and migration of embryonic neural stem cells and of cancer cells [22,23]. 

A promising strategy for anti-angiogenic cancer therapies is to antagonize Eph-ephrin interactions by interfering with the Eph receptor/ephrin system [24]. Several different synthetic peptides and small molecules have been studied for this purpose; the advantage of these artificial ligands is that they can show a much higher selectivity compared to the physiological ephrin ligands. A number of linear peptides and small molecules have been reported to act as artificial ligands of Eph receptors: KYL and other linear peptides for EphA4 [25]; TNYL-RAW peptide for EphB4 [26]; SWL and other peptides for EphA2 [27]; and dimethyl-pyrrole derivatives for EphA2 and EphA4 [28]. 

 Compared with these linear peptides, cyclic peptides show more restricted conformations that often result in higher affinity, selectivity, and stability [29,30]. Consequently, cyclic peptides are more desirable as probes of receptor functions and leads for therapeutic development. In this report, we identify a new cyclic nonapeptide, TYY, [c(CTYYWPLPC)], obtained from the M13 phage display library. TYY binds to EphA4, but not to EphB4, and selectively inhibits the binding of ligand ephrinA5 to EphA4. At a low micromolar concentration, TYY shows pronounced anti-angiogenesis activity in a 3D culture system. A molecular docking study further identifies a putative structural model for the TYY peptide bound to EphA4. This is the first cyclic peptide to be reported in the literature as a new lead for the development of conformationally restrained and enzymatically stable agents targeting EphA4.

## Materials and Methods

### Phage display

 A pre-made library consisting of a disulfide-constrained nonapeptide (Ph.D.-C7C) library (New England Biolabs, Beverly, MA) was used for panning on EphA4. Recombinant mouse EphA4 Fc Chimera protein (R&D Systems, Minneapolis, MN) was incubated overnight at 4°C in 96-well ELISA plate wells at concentrations of 10 μg/ml in coating buffer (0.1 M NaHCO_3_). Wells were blocked for 1 h with blocking buffer (0.1 M NaHCO_3_, 5 mg/ml BSA, 0.02%NaN_3_), and rinsed with TBST (0.1% Tween20 in TBS). In round 1 of panning, 5×10^10^ plaque-forming units (PFUs) of the phage library in 100 μl TBST were incubated for 1h at room temperature in EphA4 receptor-coated wells. After washing, the remaining bound phage were eluted with 100 μl of 0.2 M glycine-HCl (pH 2.2), and then neutralized by 15 μl 1M Tris-HCl (pH9.1). The entire eluate was used to infect early-log phase ER2738 host bacteria and amplified (4.5 h, 37 °C). The phage were concentrated and stored according to the manufacturer’s recommendations. In rounds 2, 3, and 4, we added 4.9×10^10^, 1.81×10^11^, 1.95×10^11^ PFUs of the amplified phage pool from the previous round, respectively, to an EphA4 Fc-coated well and a BSA coated well. The phages were panned as described for round 1, except eluted phages were tittered prior to amplification to assess enrichment.

### Phage binding assay

 Phages binding to EphA4 receptor-coated plates were quantified using an anti-phage antibody conjugated to horseradish peroxidase (M13 phage detection kit, Amersham Pharmacia Biotech, Piscataway, NJ) with 2,2-azino-bis(3-ethylbenzthiazoline-6-sulfonic acid) as substrate. A 50 μl of 4 μg/ml EphA4 Fc or 5 mg/ml BSA (as a negative control) in coating buffer (0.1 M NaHCO_3_) was incubated overnight at 4°C in 96-well ELISA plate wells. Wells were blocked for 30 minutes with blocking buffer, and rinsed with TBST. A 50 μl of amplified phage was added to each well and incubated at room temperature for 2 h. After washing with TBST, HRP-anti-M13 antibody was added to each well for 1 h and later developed with 100 μl ABTS solution (Amersham Pharmacia Biotech, Piscataway, NJ). Absorbance of each well was read at 405 nm with a Synergy 2 reader (BioTek, Synergy 2). Phages with high binding affinities were then sequenced.

### Peptide synthesis

 Two cyclic peptides, TYY and SWY, were synthesized on a Symphony peptide synthesizer (Protein technology) using Fmoc [N-(9-fluorenyl) methoxycarbonyl] chemistry. A TentaGel amide resin was used for the solid-phase synthesis. A 5-fold excess of Nα-Fmoc-amino acid, diisopropylcarbodiimide (DIC), hydroxybenzotriazole (HOBt) were used in every coupling reaction step. Removal of the N-terminal Fmoc group was accomplished by 20% piperidine in dimethylformamide (DMF). After the coupling, the resin was stirred in DMF with iodine to get cyclized peptides. The cleavage of a peptide from the resin was carried out with cleavage cocktail comprised of water (5% [vol/vol]), thiophenol (5% [vol/vol]), and triﬂuoroacetic acid (TFA) (90% [vol/vol]) for 2 h at room temperature with gentle stirring. The peptides were precipitated by adding ice-cold diethyl ether and washed repeatedly in cold diethyl ether. The crude peptides were dissolved in 20% acetonitrile in water before being lyophilized, and then were dissolved in water and purified using semi-preparative reverse-phase high-performance liquid chromatography (HPLC). The fractions containing the peptides were pooled together and lyophilized. The purity of the final products was assessed by analytical reverse-phase HPLC and by matrix-assisted laser desorption/ionization time-of-flight mass spectrometry. All of the peptides were at least 95% pure.

### ELISA-based binding assay

 For Eph receptor binding studies, 50 μl of ephrin A5 (5 μg/ml, kindly provided by Dr. Elena B. Pasquale, Sanford-Burnham Medical Research Institute) was coated onto each well on a 96-well ELISA plate (Corning, #3690) overnight at 4°C. The wells were blocked with 100 μl blocking buffer (Pierce, #37543) for 1 h and then rinsed with PBS. A 1 μg of recombinant mouse EphA4 Fc Chimera protein (R&D Systems, Minneapolis, MN) in EIA buffer (1% BSA, 0.05% Tween 20 in TBS) was then incubated in each well for 1.5 h at room temperature. The EphA4 Fc-coated wells were rinsed with PBS buffer and incubated for 1.5 h with different peptide concentrations and same amount of alkaline phosphatase (AP)-tagged ephrin A5 in a total volume of 50 μl. After the unbound peptide and ephrin had been washed away, bound ephrin-A5 AP was quantified using *p*-nitrophenylphosphate as a substrate by a Synergy2 reader (BioTek, Winooski, VT) with wavelength of 405 nm. Data were fitted using nonlinear regression. IC_50_ values were calculated using Prism (GraphPad Software Inc.).

### Human umbilical vein epithelial cell (HUVEC) viability assay and tube disruption assay

 The cytotoxic effects of the TYY cyclic peptide were measured at different concentrations by plating 1×10^4^ HUVEC cells (ATCC) per well in 96-well plates and determining cell survival. Cells were treated with various concentrations of TYY (10 μM and 20 μM) and incubated in an incubator with a 5% CO_2_ and 95% humidity atmosphere at 37°C for 24 h and 48 h. After incubation, cell viability was measured using a CellTiter-Blue assay kit (Promega) according to the manufacturer’s instructions. Briefly, 20 μl of CellTiter-Blue reagent was added to 100 μl of culture media and cells were incubated for 2-4 hours at 37°C. Afterward, fluorescence at 540_Ex_/600_Em_ was measured using a fluorescence plate reader (BioTek, Synergy 2). Each experimental data point was generated from at least three independent experiments.

 Aliquots of 300 μl Matrigel Basement Membrane Matrix (BD biosciences) were added to each well of 24-well plates, and incubated for 1 h at 37°C to allow the Matrigel to solidify. HUVECs (5×10^4^) in 500 μl endothelial cell growth medium were added to each well and incubated for 4 h at 37°C to allow the cells to form tube-like structures. The TYY cyclic peptides were diluted in DMSO to the desired concentrations (1, 5, 10 and 20 μM) and added to the cells, followed by incubation for 24 h at 37°C in a 5% CO_2_ atmosphere. A positive control was made by adding compound mHA11 [31] (1 μM) instead of TYY. Samples were examined and photographed under a fluorescence microscope (Eclipse TE2000-U, Nikon, Tokyo, Japan). The tube disruption assay was independently repeated three times for each concentration of TYY. 

### Molecular docking

 Molecular docking study of cyclic peptide TYY with EphA4 was performed using the Autodock4 program [32]. The receptor EphA4 structure at a 2.35 Å resolution was extracted from the crystal structure of the EphA4/EprinA2 complex (PDB code: 2WO3). The receptor file was converted to a PDBQT file, and a 60 × 60 × 60 grid box with a grid spacing of 0.375 Å was defined to cover the whole groove in which EphrinA2 binding site is located. The cyclic peptide TYY was constructed and following energy minimized by means of the Tripos force field and Gasteiger-Hückel charges, which were implemented in Sybyl-X1.3 [33]. The whole docking process was accomplished with Autodock4 using AutoDockTools-1.5.2.

## Results and Discussion

### Identify new cyclic peptides that bind to the EphA4 receptor

 We identified cyclic peptides that bind to EphA4 by panning an M13 phage library (consist of 10^9^ electroporated sequences) that displays random cyclic disulfide-constrained nonapeptides using the immobilized EphA4 extracellular domain fused to human Fc. The phage clones remaining bound to EphA4 were eluted with a low pH solution and amplified. After four rounds of selection on EphA4, we detected an approximately 75-fold enrichment of phage binding to EphA4. In total, 48 of the individual clones that were tested for the binding to EphA4 Fc, and 6 of them showed significant binding when compared with the BSA negative controls. All 6 of these EphA4-binding clones displayed four different cyclic peptide sequences (Table 1), and we refer to these peptides as TYY, SWY, LMT, and WPI.

**Table 1:** Sequences of 9-residue cyclic peptides identified from an M13 phage library display that show selective binding to the EphA4 receptor.

Peptide	Sequence
TYY	c[CTYYWPLPC]
SWY	c[CSWYWPLPC]
LMT	c[CLMTSQFRC]
WPI	c[CWPIKVGWC]

### The newly identified cyclic TYY selectively inhibits ephrinA5 binding to the EphA4 receptor

**Figure 1:**
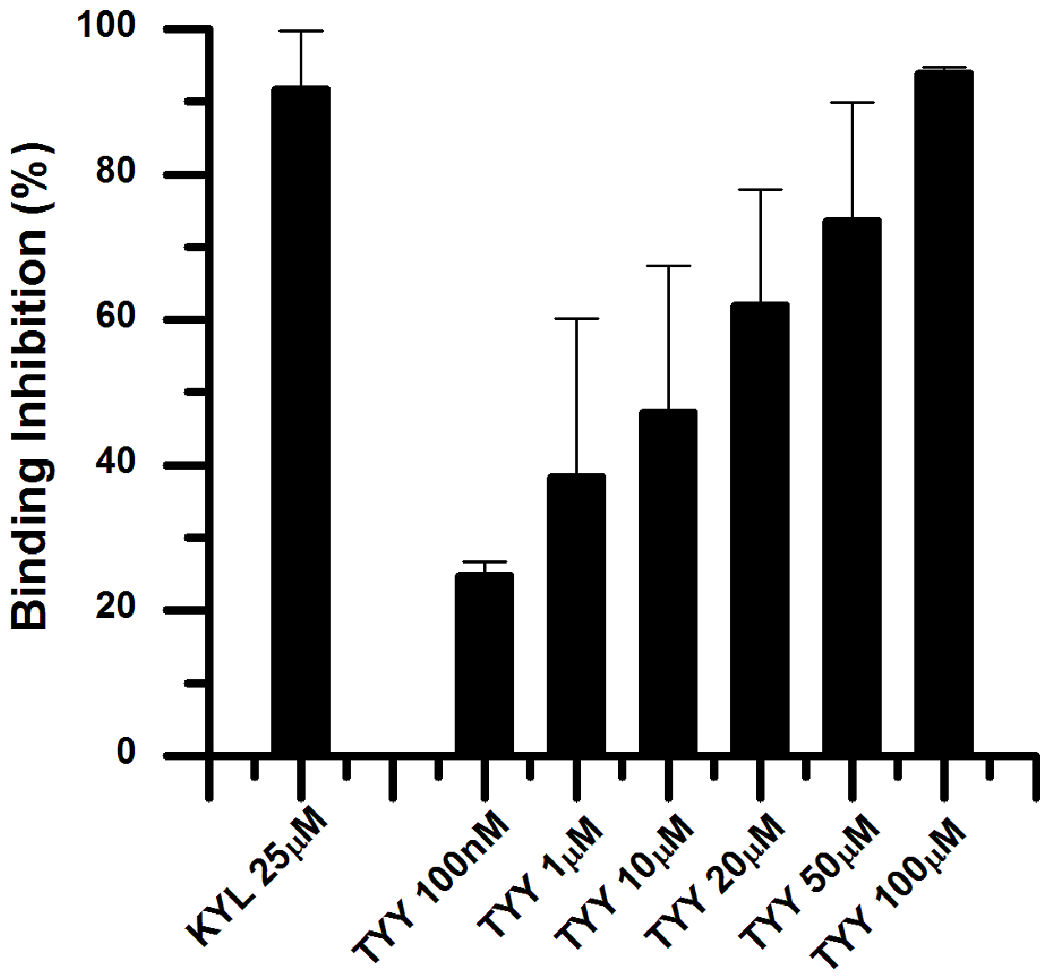
TYY cyclic peptide inhibits ephrinA5 AP binding to immobilized EphA4 Fc fusion protein. The KYL peptide was used as a positive control at a concentration of 25 μM. The histogram shows the inhibition of ephrinA5 AP bound to EphA4 in the presence of KYL or TYY at different concentrations. Error bars represent standard errors from three independent measurements.

**Figure 2:**
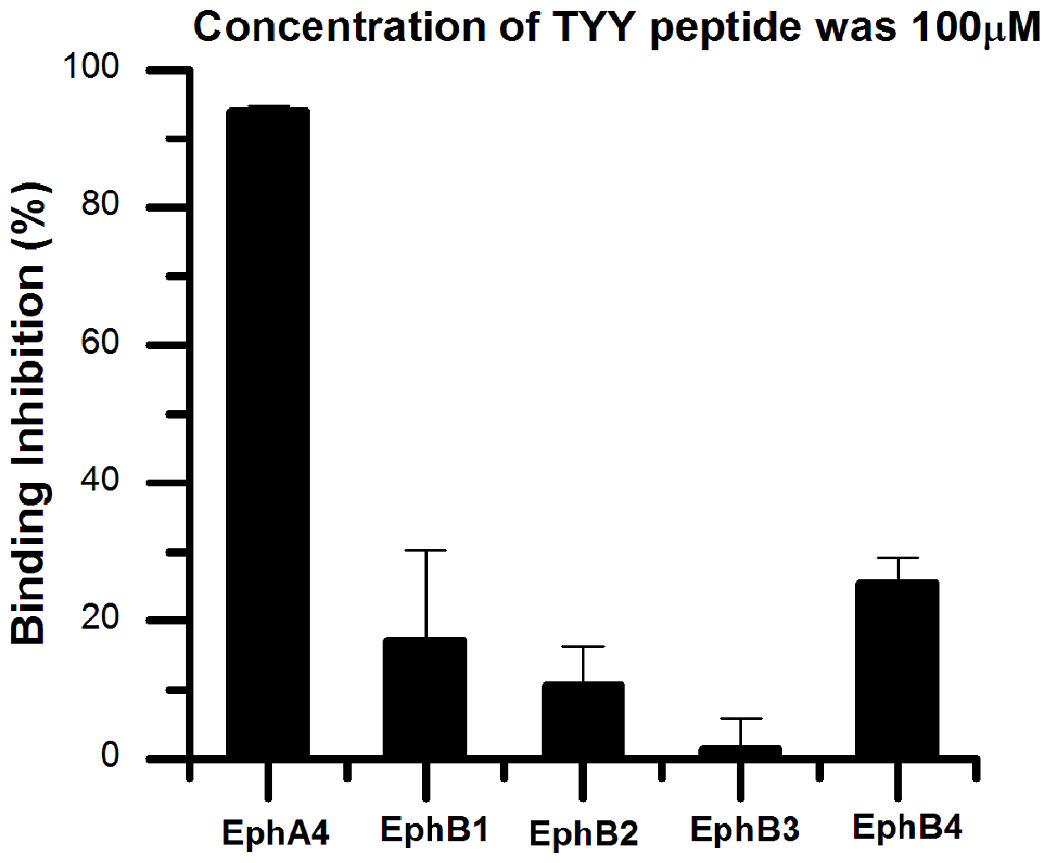
TYY cyclic peptide selectively inhibits Eph4-ephrinA5 interactions. For ephrinA5 AP binding to immobilized EphA4 Fc fusion protein, TYY shows 94% inhibition at 100 μM, but 30% or even lower inhibition for ephrinB2 AP binding to immobilized EphB receptor Fc fusion proteins (EphB1, B2, B3, and B4). The histogram shows the inhibition of each ephrin AP bound to their Eph receptors. Error bars represent standard errors from three independent measurements.

 Two cyclic peptides TYY and SWY were synthesized and purified for use in bioactivity tests. Both SWY and TYY peptides showed dose-dependent inhibition of EphA4 binding with its ligand alkaline phosphatase-tagged ephrinA5. The SWY cyclic peptide showed 40% inhibition of ephrin A5 binding to EphA4 at 50 μM, but the TYY cyclic peptide showed much better inhibition at the same concentration. The inhibition experiments indicate that TYY peptide inhibits EphA4-ephrinA5 binding by 24.8% at 100 nM and by 94.06% at 100 μM, with an IC_50_ at 11.7 μM (Figure 1). The positive control we used in the inhibition experiments was the linear 12-residue KYL peptide (KYLPYWPVLSSL) [25], which showed 92% inhibition at 25 μM. The TYY cyclic peptide at 100 μM concentration also showed significant selectivity for blocking the EphA4-ephrinA5 when compared to other EphB receptors with ephrinB2 (Figure 2). 

### Cyclic TYY effectively inhibits HUVEC tubule formation activity

**Figure 3:**
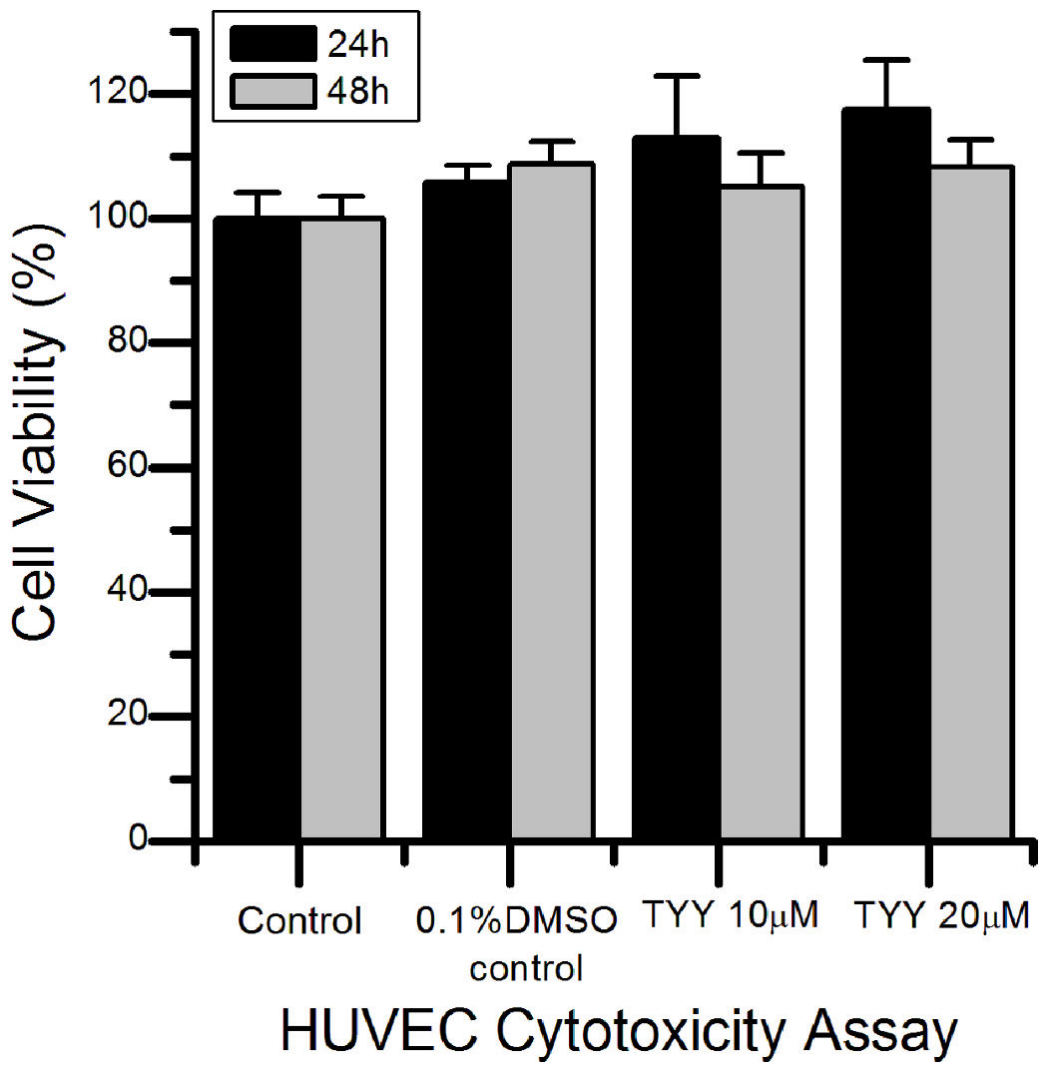
Effects of the TYY cyclic peptide on viability of HUVEC cells. The HUVECs were treated with the indicated concentrations of the TYY peptide. A 0.1% DMSO control was used because the highest concentration of the DMSO vehicle in the TYY solutions was 0.1%. The cell viability was determined by CellTiter-Blue assays 24 h and 48 h after addition of TYY. Each experimental data point was generated from at least three independent experiments.

**Figure 4:**
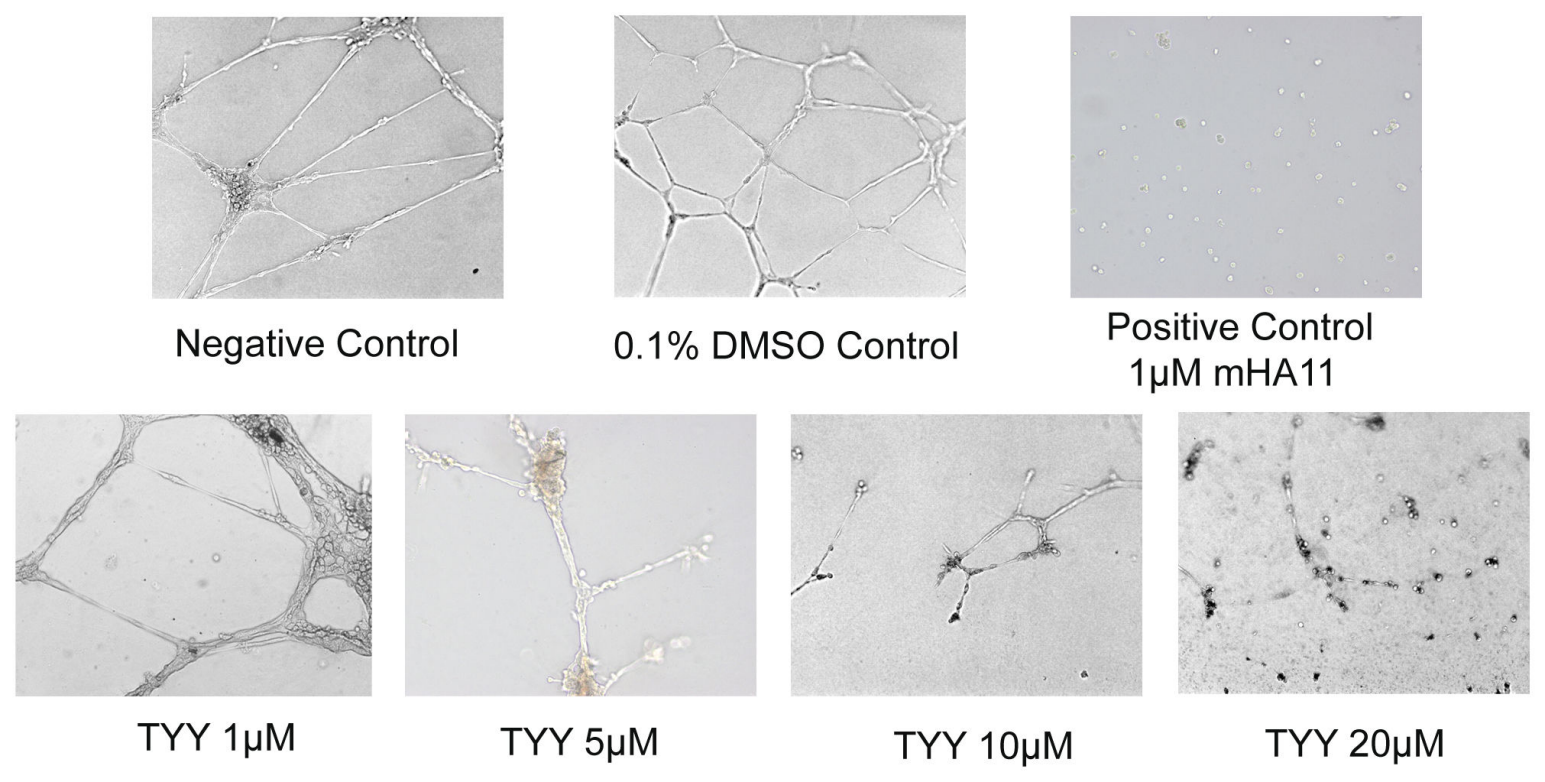
Effects of the TYY cyclic peptide on *in* *vitro* vascular tube formation. Spontaneous formation of capillary-like structures by HUVECs on Matrigel was used to assess the potential of TYY for inhibition of vascular tube formation. Upper panel: Untreated negative control and vehicle (0.1% DMSO) treated HUVEC control showed capillary-like structures after incubation of HUVECs for 24 h. A positive control (1 μM mHA11) showed complete inhibition of HUVEC-tube formation. Lower panel: Effects of different concentrations (1, 5, 10, and 20 μM) of TYY on HUVEC tube formation.

 The TYY cyclic peptide was evaluated for its cytotoxic effects on HUVECs. After exposure to 10 and 20 μM TYY for 24 h and 48 h, HUVEC viabilities were assessed by the CellTiter-Blue cell viability assay. As shown in Figure 3, TYY had no cytotoxic effects on HUVECs after 48 h at either 10 or 20 μM. The TYY cyclic peptide was then assessed for its effects on *in vitro* HUVEC tubule formation. The HUVECs showed the ability to form tubular structures and meshes of capillary-like vessels when plated on a Matrigel matrix for 24 h. We used this *in vitro* model to evaluate the anti-angiogenesis activity of the TYY cyclic peptide. The inhibition of HUVEC tube formation by the TYY cyclic peptide is shown in Figure 4. A negative vehicle control (0.1% DMSO) showed no inhibitory effects on the tube formation activity of HUVECs. However, the positive control (mHA11) exhibited complete inhibition. The inhibitory activity of TYY on HUVEC tube formation was first evident at 5 μM and complete inhibition was observed at 20 μM; this response was comparable to that of the positive control (mHA11). We performed HUVEC viability assay and confirmed that TYY at concentrations up to 20 μM showed no cytotoxic effects in the HUVECs. These results confirmed that the anti-angiogenesis activity of TYY was not caused by cell death. Therefore, TYY is a desirable new candidate inhibitor of EphA4 that targets angiogenesis and merits further modification and evaluation. 

### Molecular docking reveals that the TYY cyclic peptide occupies the same binding site on EphA4 where the natural ephrin ligand binds

 The structure of the EphA4 ligand-binding domain in complex with ehprinA2 was determined by X-ray crystallography (PDB code: 2WO3), revealing the molecular interactions in the high-affinity interface between the ephrinA2 G-H loop and the EphA4 hydrophobic channel [34]. Residues Arg162, Thr104, Arg106 and Glu55 of EphA4 also showed hydrogen bonds interactions with ephrinA2 residues on the G-H loop. The TYY cyclic peptide appears to inhibit the binding of the ephrin A5 ligand to the EphA4 receptor, which suggests that the TYY peptide binds to the same pocket that the ephrin ligand binds to EphA4. Based on this information, we used Autodock4 to perform a docking study of the interaction between the TYY peptide and EphA4 by defining the box that covered the whole groove in which the ephrinA2 binding site was located. The estimated binding free energy between the TYY peptide and EphA4 as determined by Autodock4, was -6.21 kcal/mol, and the predicted *K*_*i*_ was 28.2 μM. 

**Figure 5:**
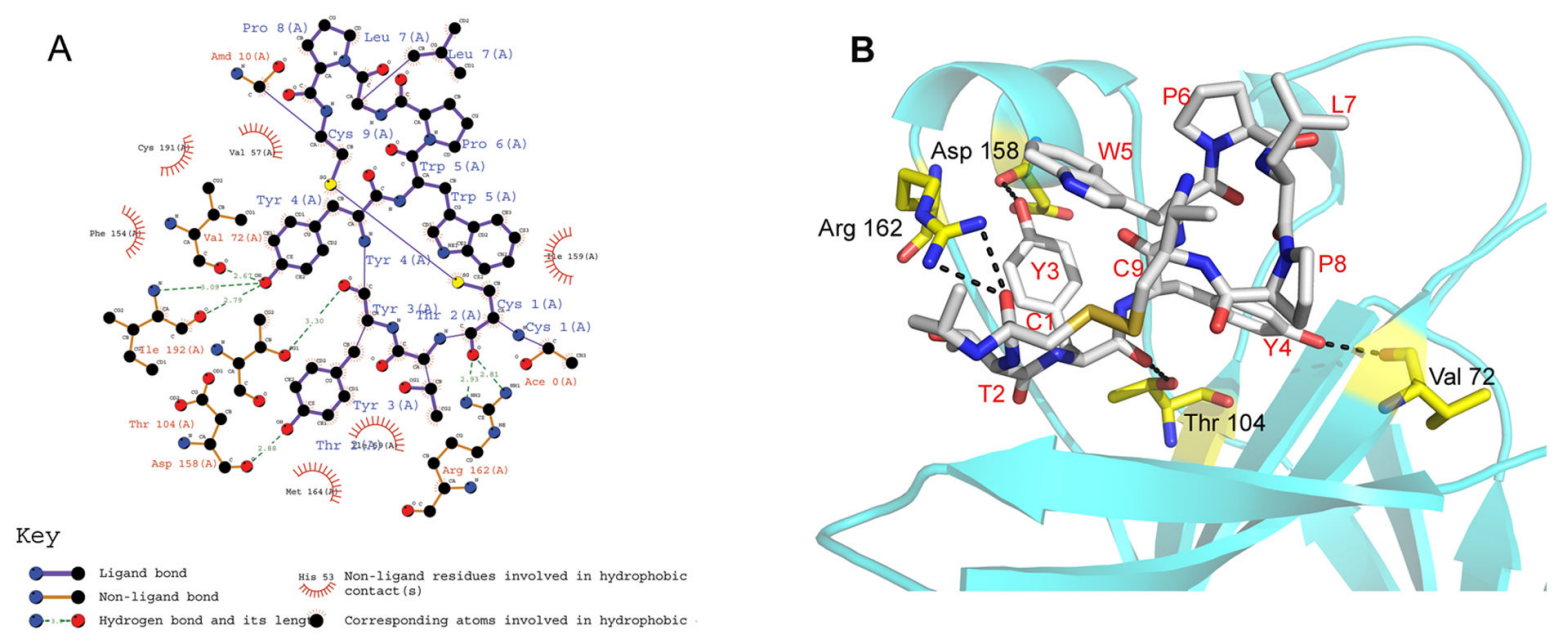
Binding mode of TYY with EphA4 receptor. (A) 2D interactions between the TYY cyclic peptide and the EphA4 receptor (by LigPlot). Residues with purple bonds in blue labels are from the ligand TYY peptide; residues with brown bonds in red labels are from EphA4. The green dashed line represents a potential hydrogen bond and its length. (B) 3D interactions between the TYY cyclic peptide and the EphA4 receptor (by Pymol). This model was generated by Autodock4, based on the crystal structure of EphA4 in complex with ephrinA2 (PDB: 2WO3). The TYY cyclic peptide is shown as a stick model with grey representing carbon atoms, red representing oxygen atoms and blue representing nitrogen atoms. The EphA4 is shown as a cyan ribbon with key residues shown as yellow sticks. Black dashed lines represent the hydrogen bonds.

**Figure 6:**
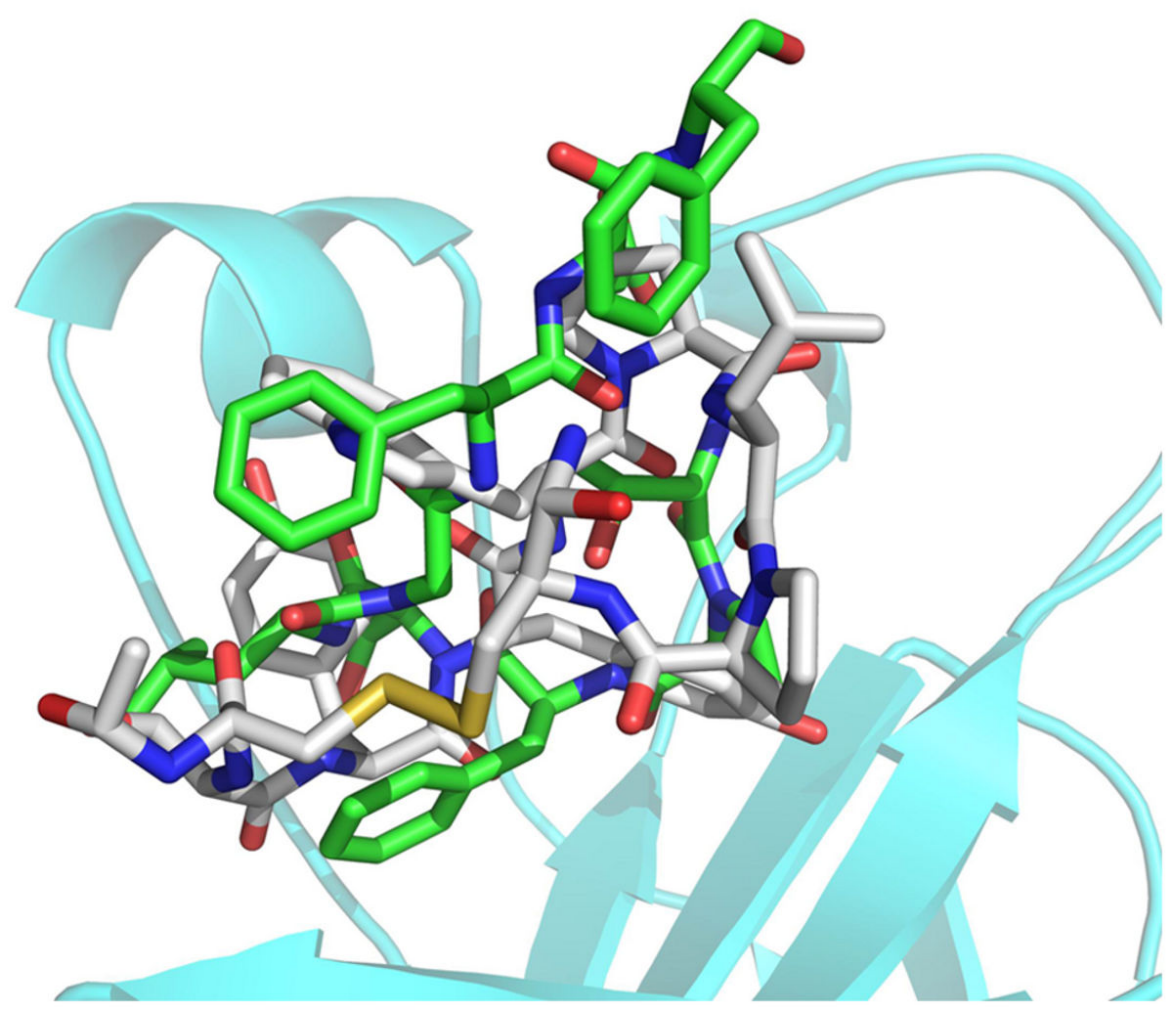
Superposition of the TYY cyclic peptide with the ephrinA2 G-H loop region from the crystal structure of its complex with the EphA4 receptor (by Pymol, PDB: 2WO3). The TYY cyclic peptide is shown as a grey stick model, and ephrinA2 G-H loop region is shown as green stick model with red representing oxygen atoms and blue representing nitrogen atoms. The EphA4 is shown as a cyan ribbon.

 As shown in Figure 5A and 5B, the Tyr3 side chain of TYY formed a hydrogen bond with the EhpA4 Asp158 backbone carbonyl group at 2.88 Å. At the same time; the Tyr3 main chain oxygen atom also formed a hydrogen bond with the EphA4 Thr104 backbone at 3.30 Å. Comparison with the SWY peptide sequence and activity indicated that the Tyr3 on TYY may play an important role in the interaction with EphA4. For Tyr4 on the TYY peptide, the side chain hydroxyl group potentially formed a hydrogen bond with the EphA4 Val72 or Ile192 main chain oxygen. Cys1 of TYY contributed another hydrogen bond interaction with one of the key EphA4 residues, the Arg162 guanidinium group. Hydrophobic interactions were also the major interactions between TYY and EphA4, because most of the TYY residues were highly hydrophobic. Superposition of the TYY cyclic peptide with the ephrinA2 obtained from the crystal structure of its complex with EphA4 (Figure 6) showed that TYY occupied the same groove where the ephrinA2 G-H loop binds. 

## Conclusion

 We have discovered and characterized a new cyclic peptide TYY (c[CTYYWPLPC]) that selectively binds to the EphA4 receptor and inhibits the binding of ligand ephrin-A5 to EphA4. Treatments of human HUVEC cells with low micromolar concentrations of TYY for 24 h significantly inhibited the vascular formation activity of these cells. Cell viability assays indicated that TYY’s anti-angiogenesis effects, indicated by the disruption of the formation of tubular structures, were not due to lethal effects on the HUVECs. A molecular docking study further indicated that TYY recognizes the same binding pocket on EphA4 that the natural ephrin ligand binds to and that Tyr3 and Tyr4 are important for the TYY/EphA4 interaction. These results demonstrate that TYY is a promising new lead and that worth for further modification and development will be worthwhile for establishing more effective therapeutics that target EphA4, a receptor that is widely expressed in cancer cells and tumor vasculature. 
